# Comparison of computerized tomography and ultrasound for diagnosing soft tissue abscesses

**DOI:** 10.1186/2036-7902-4-5

**Published:** 2012-04-17

**Authors:** Romolo Gaspari, Matt Dayno, Justin Briones, David Blehar

**Affiliations:** 1 Department of Emergency Medicine, University of Massachusetts University Hospital, 55 Lake Ave North Worcester, MA, 01655, USA

**Keywords:** Abscess, ultrasound, skin and soft tissue infection, cellulitis, superficial, computerized tomography

## Abstract

**Background:**

The diagnosis of a superficial abscess is usually obtained through history and physical exam but bedside ultrasound (US) and computerized tomography (CT) are sometimes used to assist in the diagnosis. It is unclear which imaging modality is superior for patients with superficial soft tissue infections. We compared the diagnostic accuracy of CT and US in patients with skin and soft tissue infections.

**Methods:**

Patients presenting with a suspected skin abscess that underwent both US and CT imaging were eligible for inclusion. Two physicians blinded to patient characteristics and other imaging results prospectively reviewed the CT and US images for pre-defined image elements, and in circumstances where there was disagreement between these interpretations, a third physician adjudicated the findings. The presence or absence of an abscess cavity was noted on imaging. Imaging detail was summarized using a pre-specified 4-point scale based on the degree of visible detail with higher numbers corresponding to greater detail. The clinical presence of an abscess was defined by surgical evacuation of purulence. Sensitivity and specificity for both CT and US were calculated using Chi square analysis. Comparison between imaging detail was performed using a Student's T-test. Data are presented with (95% confidence intervals) unless otherwise noted.

**Results:**

Over an 18 month period 612 patients received a soft tissue bedside ultrasound with 65 of those patients receiving a CT for the same complaint. 30 of these 65 patients had an abscess located in the head and neck (37%), buttock (17%), lower extremity (17%), upper extremity (13%), torso (13%), or hand (3%). US demonstrated a sensitivity and specificity for the diagnosis of abscess of 96.7% (87.0% to 99.4%) and 85.7% (77.4% to 88.0%) respectively. The overall sensitivity and specificity of CT for the diagnosis of an abscess was 76.7% (65.5% to 82.8%) and 91.4% (81.8% to 96.7%) respectively Overall image detail ratings were superior for US compared to CT (3.5 vs 2.3, p = 0.0001).

**Conclusion:**

US is more sensitive then CT, but CT is more specific for superficial soft tissue abscesses. US demonstrated more visible detail within the abscess cavity compared to CT.

## Background

The incidence of skin and soft tissue infections in the ambulatory setting has more than doubled over a ten-year period to 3.4 million emergency department visits in 2005 [1]. The epidemiology of skin and soft tissue infections has also changed due to the emergence of community-associated methicillin-resistant *Staphylococcus aureus *[2,3]. The diagnosis of an abscess is usually obtained through history and physical examination but occasionally, imaging techniques such as computerized tomography (CT) or ultrasound (US) are used to assist in the diagnosis. Differentiating a focal collection of purulence (abscess) from diffuse infection of the skin (cellulitis) is critical, as the former is treated with incision and drainage (I and D) and the latter is treated with antibiotics alone.

It is unclear which imaging modality is superior for patients with superficial soft tissue infections. CT provides excellent detail of soft tissue and is available in most hospitals in the United States but requires ionizing radiation which can be more difficult to obtain and usually involves intravenous contrast [4]. US is commonly available and provides detailed imaging of superficial structures without radiation or contrast agents. US is becoming a common initial imaging test for suspected skin abscesses, but the relative merits of US compared to CT have not been explored [5]. We compared the diagnostic accuracy of CT and US in patients with skin and soft tissue infections.

## Methods

This study was a retrospective review of patients with bedside US for a suspected skin abscess presenting to an urban academic emergency department over an 18-month period. Patients were eligible for enrollment if they had localized swelling, pain, induration, and warmth suspicious for a soft tissue abscess and underwent both US and CT scan of the affected area. Patient care was provided by emergency medicine residents working with emergency medicine attending faculty. Patients were excluded if the image sets for either US or CT were incomplete. US imaging was performed prior to CT imaging in 91% of patients. This study was approved by our institutional review board with waiver of informed consent.

### Computerized tomography

CT examinations were performed at presentation using a Brilliance 64 slice CT scanner (Phillips Healthcare, Andover, MA, USA). Images were obtained following the institutional protocol for soft tissue imaging, including intravenous contrast for patients with appropriate renal function. CT images were acquired centered on the body area of interest with a slice thickness between 2 and 4 mm. The direct multiplanar reformation function was used to generate coronal and sagittal reformations with a slice thickness between 2 and 4 mm. The attending physician interpreting the CT images was not blinded to the US results but is not routinely given this information. Interpretation of the presence or absence of an abscess on CT imaging was determined using the final interpretation in the patient record. No patients in this study had a change in the interpretation of the CT findings.

### Sonographic imaging

US imaging was performed at presentation using a Zonare ultrasound machine (Zonare Inc., Mountain View, CA, USA) with a 7.5-10 MHz high frequency linear array transducer. Sonographers included residents and faculty with > 25 soft tissue ultrasounds. Standard images were obtained of suspected soft tissue abscess collections, and a complete image set was defined as long and transverse B-mode images of the abscess cavity and surrounding soft tissue, and images of a contralateral anatomic site for comparison. All US images were recorded as video images. The US images were categorized as demonstrating an abscess cavity or not using an adjudication process involving two to three physicians. Two physicians with experience in soft tissue US were blinded to patient characteristics, and the results of the CT reviewed the US images for the presence or absence of an abscess. In circumstances where there was a disagreement between these interpretations, a third physician adjudicated the findings by reviewing the images and the reasoning of initial interpretations. All three reviewers have at least 5 years of experience in emergency ultrasound with more than 300 soft tissue ultrasounds. The most experienced reviewer has over 10 years of experience with greater than 700 soft tissue ultrasounds.

### Image detail ratings

**Figure 1:**
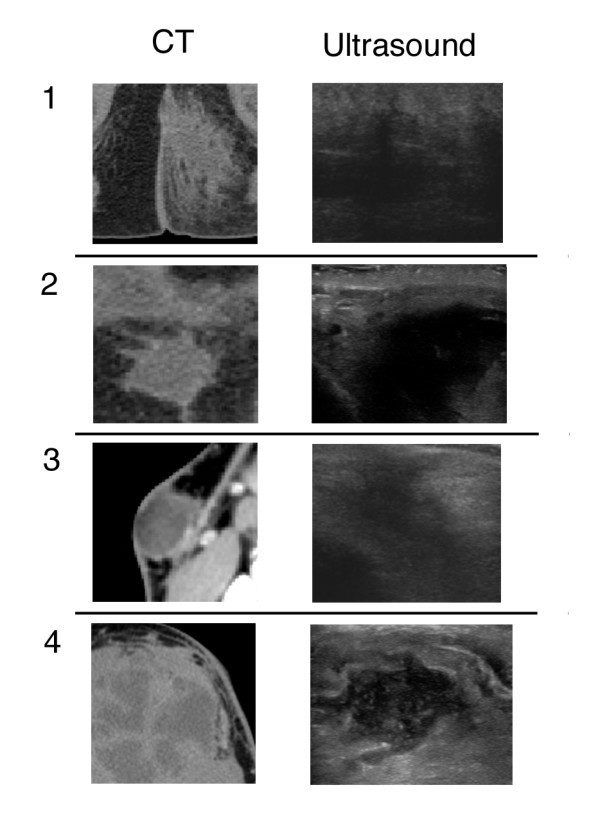
**Imaging of abscess cavity debris rating**. The degree of visualized detail for both US and CT images was rated using a 4-point scale. A rating of 1 corresponded to visualized changes in the soft tissue but no organized fluid collection. A rating of 2 corresponded to visualization of a discrete abscess cavity but no internal details (i.e. a homogenous center). A rating of 3 corresponded with visualization of heterogeneous contents of the abscess cavity without fine detail, and a rating of 4 corresponded with visualized fine details within the abscess cavity.

CT and US images were evaluated to determine the level of detail provided by each imaging modality using a predetermined set of criteria based on the amount of detail visible in the abscess cavity. Images were categorized as demonstrating any details within the abscess cavity or not. If abscess cavity contents were visible, the contents were further characterized as demonstrating fine details or not. Imaging was summarized using a four-point scale based on the degree of visible detail with higher numbers corresponding to greater detail (Figure 1). Both CT and US findings were determined using the same adjudication process described above. All interpretations of the image detail were performed by physicians who were blinded to patient characteristics and the results of alternative imaging.

### Final diagnoses

The criterion standard for final diagnosis of an abscess was determined using documentation of evidence of abscess purulence using a structured electronic medical record review as described previously [6]. Briefly, each record was initially reviewed in a structured format by a single physician to categorize each patient into one of two categories, "abscess" or "no abscess" based on documentation in the chart. The final diagnosis of abscess was defined by either (1) the documented presence of purulence following surgical drainage or (2) documented results of culture of abscess purulence. All patients who did not meet the above criteria were defined as 'no abscess' for the purpose of this study. As this study is a retrospective review; no patients were contacted to confirm the presence or absence of an abscess.

If the initial categorization using the above criteria matched the final discharge diagnosis, then this was considered as the final diagnosis for the purpose of this study. If there was a disagreement, a second independent investigator reviewed the patient's electronic medical record using the same guidelines for determining final diagnosis. If two of the three final diagnoses agreed, this was considered the final diagnosis. To limit misclassification and to allow for information to be posted to the electronic medical record, the record was reviewed at least 3 months following the initial encounter. The 3-month time frame was to assure that all potential clinical follow-up visits were captured and to allow for delay in posting to the electronic medical record.

Statistics-mean values were calculated and presented with 95% confidence intervals. A Fischer's exact test was used to determine the degree of association between CT interpretation and US interpretation. Sensitivity, specificity, and accuracy were calculated for each test (US and CT) to diagnose the presence or abscess of an abscess cavity. Comparison between groups was performed using a Student's *t *test (continuous data) or Fisher's exact test (categorical data).

## Results and discussion

A total of 612 patients received a soft tissue US over 18 months for a suspected abscess or infected fluid collection. Sixty eight of these patients also received a CT for the same complaint during the same emergency department visit, but three of these patients were excluded due to incomplete imaging of the abscess cavity. Forty-one percent of the patients with both imaging modalities were female and the average age was 41.7 years (± 16.2). Of the patients included in this study, 46.1% were ultimately diagnosed with an abscess. The location of the abscess covered a range of anatomical locations (see Table 1). See Table 2 for the final discharge diagnoses for all patients.

**Table 1:** Anatomic locations of abscesses

	Number (percentage)
Head and neck	11 (37)
Buttock	5 (17)
Lower extremity	5 (17)
Torso	4 (13)
Upper extremity	4 (13)
Hand	1 (3)

**Table 2:** Final diagnoses of patients receiving imaging for suspected soft tissue abscess

Diagnosis	Number of patients	Percent of total patients
Abscess	30	46.2
Cellulitis	12	18.5
Other soft tissue infection	5	7.7
Soft tissue mass	4	6.2
Post surgical changes	5	7.7
Hematoma	3	4.6
Hernia	2	3.1
Other	4	6.2

**Figure 2:**
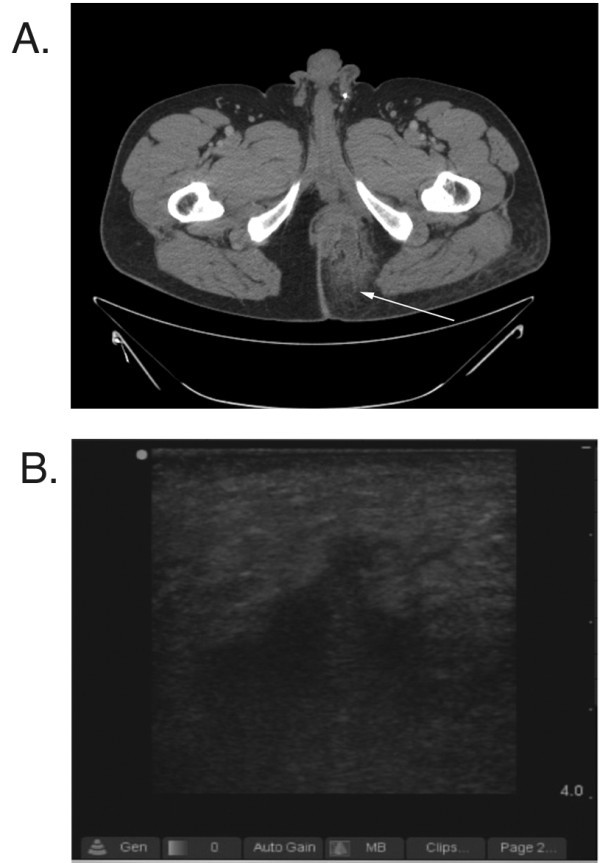
**US and CT of patient with perirectal abscess**. Figure (**A**) demonstrates CT imaging of a patient with a perirectal abscess. Note the stranding in the soft tissue (white arrow) but lack of visualized abscess cavity as interpreted by the original radiologist, and (**B**) demonstrates sonographic imaging of the same patient (long axis orientation) with characteristic loss of soft tissue planes superior and lateral to an anechoic abscess cavity. An extension of the abscess cavity "points" towards the skin surface.

**Figure 3:**
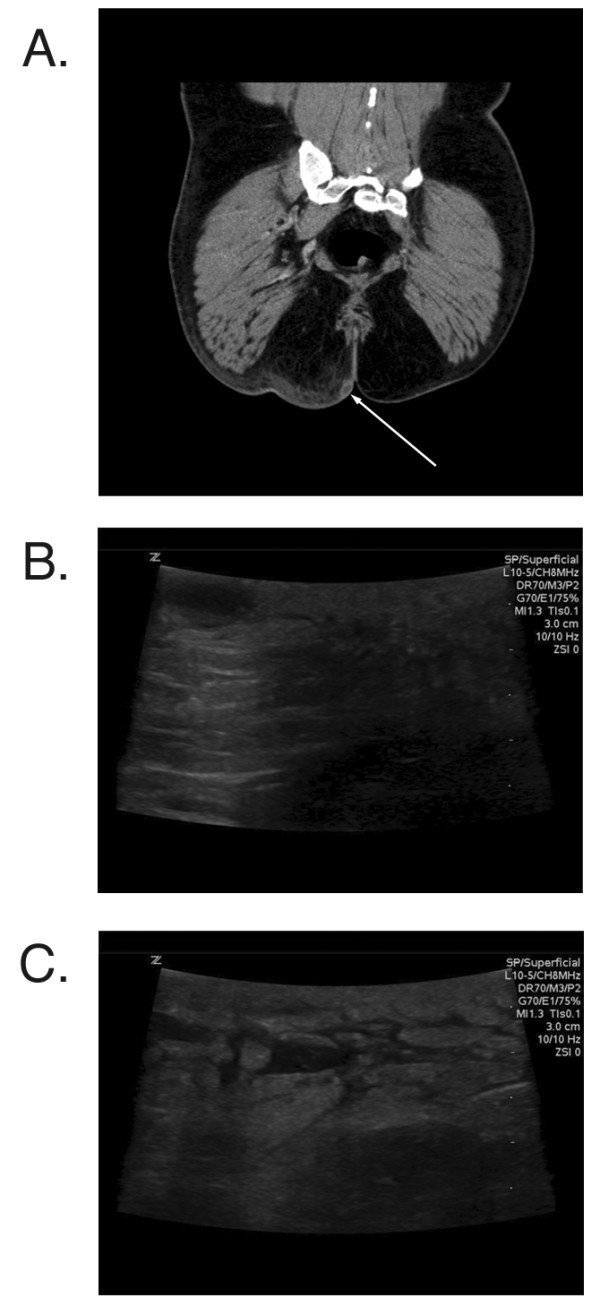
**US and CT of patient with buttock abscess**. Figure (**A**) demonstrates CT imaging of a patient with a buttock abscess. Note the stranding in the soft tissue with small central abscess cavity (white arrow). (**B**) demonstrates sonographic imaging of the same patient (long axis orientation) with visualized debris in the dependent portion of the abscess cavity and posterior enhancement deep to the abscess cavity. (**C**) demonstrates sonographic imaging of tissue lateral to the abscess cavity with branching anechoic wisps surrounding small islands of hyperechoic tissue.

CT correctly diagnosed the presence of an abscess in 23 of 30 patients and correctly diagnosed the lack of an abscess in 32 of 35 patients. Figure 2 includes example images from a patient where the abscess cavity was not visualized by CT. The overall sensitivity and specificity of CT for the diagnosis of an abscess was 76.7% (65.5% to 82.8%) and 91.4% (81.8% to 96.7%) respectively. Additional information provided by CT that related to the extent of the abscess cavity included extension of the abscess cavity to the retroperitoneum (one patient), extension of the infection to a bone or joint (two patients), and presence of muscle destruction and gas in the tissue (one patient). US correctly diagnosed the presence of an abscess in 29 of 30 patients and correctly diagnosed no abscess in 30 of 35 patients with an alternative diagnosis. Figure 3 demonstrates images from a patient where the abscess was visualized by both CT and US. The overall sensitivity and specificity of US for the detection of an abscess was 96.7% (87.0% to 99.4%) and 85.7% (77.4% to 88.0%) respectively. There was no statistical difference in the accuracy between US and CT (90.8% vs. 84.6%). In addition, there was no difference in the accuracy between USs performed by attending physicians and residents (*p *= 1.000).

Not all patients received i.v. contrast during the CT imaging. Those who underwent CT imaging with i.v. contrast received an average of 97 cc of Isovue 370 contrast (Bracco Diagnostics Inc., Princeton, NJ, USA). A total of nine patients did not receive i.v. contrast, but there was no difference in diagnostic accuracy between CT scans with or without i.v. contrast. Eight of the 9 patients (88.9%) without i.v. contrast were correctly diagnosed compared to 50 of 56 patients (89.3%) who received i.v. contrast.

**Figure 4:**
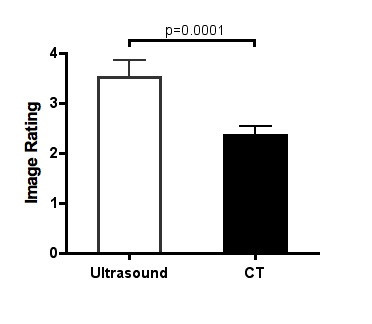
**Image rating for ultrasound and CT images of patients with abscess cavities**. Figure 4 compares the average (95% confidence interval) rating for ultrasound and CT images of patients with a superficial abscess cavity. Images were rated on the following numeric scale from 1 to 4 based on image details with 1 corresponding to changes in the soft tissue but no abscess cavity visualized and 4 corresponding to fine details of the abscess cavity contents visualized.

In the patients with abscesses, US imaging provided superior fine imaging detail compared to CT. Of the 30 patients with an abscess, three of the patients demonstrated US images where the internal details of the abscess cavity were not visible, and 26 demonstrated images with visible contents within the abscess cavity. The agreement between reviewers for US image rating system demonstrated a kappa value of 0.20. CT images for these same 30 patients demonstrated 4 patients with no discrete details of the abscess cavity contents and 15 with some visualization of the abscess cavity contents. The agreement between reviewers for CT image rating system demonstrated a kappa value of 0.34. Of the patients where the contents of the abscess cavity was visualized, US visualized fine details of the debris in 20 of 26, compared to 3 of 15 for CT. Image detail ratings were superior for US compared to CT (Figure 4).

The results of this study demonstrate that both CT and US accurately identify superficial soft tissue abscesses, but US demonstrated a superior sensitivity and CT demonstrated a superior specificity. The question of how to integrate CT and US into the care of patients with suspected superficial soft tissue infections is a matter of debate, but the role of an imaging modality in these patients is not dictated solely by the sensitivity and specificity of the diagnostic modality. CT provides details concerning distant structures such as underlying bone, muscle and organs that may not be visualized during sonographic imaging due to the limited depth imaged by ultrasound. In contrast, US is a dynamic imaging modality that provides information not available by CT, such as streaming of abscess purulence with manual pressure or the ability to dynamically guide surgical drainage. Other aspects of US such as the use of Doppler imaging have also been shown to be useful in diagnosing superficial abscesses [7]. It is not clear why ultrasound was more sensitive than CT for soft tissue ultrasound, but we speculate that this is due to the ability of ultrasound to provide greater submillimeter detail combined with the ability of ultrasound to provide dynamic imaging. The higher sensitivity of US relative to CT supports the use of US as the initial imaging modality. CT can be reserved for cases where either the US images are unclear or the abscess cavity extends into deeper tissue planes.

Diagnostic imaging is not required for all superficial abscesses but in some clinical situations, it can allow more informed decisions and support better patient care. Recent literature on the use of US in superficial soft tissue infection is starting to better delineate the role of US in this patient population, but corresponding literature in CT is lacking [5,8,9]. Radiation exposure, cost, and intravenous contrast may limit the use of CT to specific types of soft tissue infections, such as infections with potential extension into deeper tissues or those with suspected complications such as osteomylitis. In contrast, the lack of radiation, ability to provide dynamic guidance during surgical drainage, and portability of US make it an ideal imaging modality for soft tissue infections. However, if US is to become more fully integrated into the care of superficial soft tissue infections, then a number of questions remain to be answered regarding the best practice for the evaluation and care of these patients.

It is not clear if the level of detail visualized by US is clinically important, as relatively little research has been done to characterize the different sonographic features of soft tissue infections or to determine the clinical relevance of those findings. A few researchers have attempted to categorize soft tissue infections using basic sonographic features. Preliminary data on sonographic features of cellulitis demonstrate some characteristics that may predict outcomes [10]. Chao et al categorized the range of infection from cellulitis to abscess cavity into four categories (1) subcutaneous thickening without disarray or pus accumulation, (2) disarray of subcutaneous tissues without pus accumulation, (3) disarray of subcutaneous tissues with pus accumulation, and (4) abscess formation but did not discuss sonographic findings within the abscess cavity [11]. Tiu et al characterized breast abscesses by echogenicity, contour of the abscess cavity, and the presence of absence of various features such as a hypoechoic rim, posterior enhancement, and others [12]. The majority of research focusing on skin and soft tissue infections use more simplified descriptions of their findings and focus on echogenicity and shape of the abscess cavity [13-16]. A detailed, comprehensive classification system for skin and soft tissue abscesses is lacking but would be useful to help structure future research. Research focusing on the association of sonographic findings with clinical outcomes would be useful to help guide the clinical care of patients with soft tissue infections.

## Conclusions

In summary, our findings provide preliminary data that US is more sensitive than CT for diagnosing skin and soft tissue infections but CT is more specific. Ultrasound provides more detailed information concerning the abscess cavity without ionizing radiation but the clinical importance of this is unknown. Further research is needed to determine how to best use both US and CT in patients with skin and soft tissue infections.

### Limitations

This study was a retrospective study and suffers from the limitations of all retrospective studies such as a reliance on the accuracy of the written record and dealing with missing data. This study suffers from significant selection bias as only 65 of the 612 patients presenting with complaints suspicious for an abscess were included. This decreases the clinical implications of this study but does not eliminate the finding that in some patients, US demonstrated an abscess when CT did not. It is likely that the utility of US in the majority of patients that did not receive CT was equal to the patients included in this manuscript, as US has been demonstrated to be useful in less complicated patients that rarely receive CT imaging.

Blinding was unbalanced as only the individuals interpreting the US were blinded to the results of the CT. There were limitations related to blinding, as the individuals interpreting the CT were not blinded to the results of the US. In addition, the designation of no abscess is limited by the retrospective nature of this study, and it is possible that some patients categorized as no abscess, in fact, had an abscess that resolved spontaneously. This would result in a reduction of specificity but would have no effect on sensitivity. Finally, the overall number of patients with an abscess was low.

## Competing interests

The authors declare that they have no competing interests.

## Authors' contributions

RJG designed the study, MD and JB assisted in image capture and database design. RJG and DB performed US image analysis. MD and JB performed CT image analysis. RJG performed data analysis and statistics. All authors read and approved the final manuscript.
